# Misinnervation in the third nerve palsy: Vertical synergistic divergence or consummate congenital bilateral asymmetrical Brown’s syndrome with congenital ptosis?

**DOI:** 10.4103/0301-4738.71696

**Published:** 2010

**Authors:** Pramod Kumar Pandey, Subhash Dadeya, Anupam Singh, Pankaj Vats, Neha Rathi, Sonal Dangta

**Affiliations:** Guru Nanak Eye Center, Maulana Azad Medical College, University of Delhi, New Delhi, India

Dear Editor,

We read with interest the report by Jethani.[1] The author propounds vertical synergistic divergence in congenital third nerve palsy, without authenticating either; instead the child displays seminal motility findings of a typical congenital asymmetrical bilateral Brown’s Syndrome [BS], right > left, with cognate congenital ptosis and hypotropia of the right eye. The ocular motility is not even remotely in concordance with third nerve palsy [unilateral or bilateral] or with attendant aberrant innervation as educed by the author. Jaw winking phenomena if present would have clinched the issue in favour of congenital ptosis, but this was not tested.

Ocular motility conforms to the pattern of alternating abducting hypertropia, broadly sported by bilateral primary superior oblique overactions [PSOOA], bilateral inferior oblique / inferior rectus palsies [IOPs / IRPs], bilateral BS, and laterally alternating skew. A negative head tilt test rules out bilateral IOPs and IRPs. Bilateral PSOOA will not produce primary position deviation or ptosis and ductions will be full, congenital laterally alternating skews are non-isolated. The jigsaw for bilateral BS is complete.

Widening of a palpebral fissure and downshoot of the eye in adduction may be seen in BS, but they have been interpreted as lid retraction and synergistic divergence by the author.[1,2] Bilateral cases usually sport a V pattern, provided elevation is possible, and an A pattern noted as superior obliques [SOs] are overacting.[1] BS may not have intorsion in the primary position, but as the eyes elevate intorsion sets in.

Underaction of the IOs in the ductions was noted[1] and was strongly suggestive of BS. A forced duction test holds the key, but was overlooked. SOs could be underacting / normally acting or overacting in BS and could influence the surgical strategy, as SO tenotomies with overacting SOs might not end up in SO palsies postoperatively.

Brown’s Syndrome is rare, being bilateral in 5 to 10% of the cases.[2] Associations include congenital ptosis and Marcus Gunn Jaw winking phenomena,[2] all clubbed under congenital cranial dysinnervation disorders [CCDDs],[3] congenital third nerve palsy is not one of them, as surmised.[1] Right-sided preponderance in unilateral cases may explain more severe involvement with ptosis and hypotropia in the right eye, here. An autosomal dominant mode of inheritance, with incomplete penetrance and variable expression has been reported.[2] Literature is scant on bilateral BS, be it the spectrum of presentation, diagnostic dilemmas or management paradigms. An uncontrolled bilateral SO weakening procedure may generate an esoshift in the primary position, notwithstanding the fact that even SO tenectomies may not restore elevation in such severe cases. BS is only relieved, not cured, and such severe cases pose formidable surgical challenges. The findings cohere quintessentially to bilateral BS.[1] and not misinnervation in third nerve palsy as proposed by the author.
